# National age-of-consent laws and adolescent HIV testing in sub-Saharan Africa: a propensity-score matched study

**DOI:** 10.2471/BLT.18.212993

**Published:** 2018-11-20

**Authors:** Britt McKinnon, Ashley Vandermorris

**Affiliations:** a Centre for Global Child Health, The Hospital for Sick Children, 525 University Ave., Suite 702, ON M5G 2L3 Toronto, Canada.; b Division of Adolescent Medicine, Hospital for Sick Children, Toronto, Canada.

## Abstract

**Objective:**

To estimate the association between legal age of consent and coverage of human immunodeficiency virus (HIV) testing among adolescents in countries with high HIV-burden.

**Methods:**

We analysed data from adolescents aged 15–18 years, who participated in Demographic and Health Surveys or AIDS Indicator Surveys between 2011 and 2016, in 15 sub-Saharan African countries. To improve balance in the distribution of measured individual- and country-level characteristics, we used propensity score matching between adolescents in countries with more versus less restrictive age-of-consent laws (≤ 15 years versus ≥ 16 years). We estimated the percentage of individuals who self-reported that they have done an HIV test in the past 12 months and compared the differences in such testing rates among adolescents exposed to lower versus higher age-of-consent laws. We also investigated effect modifications by sex and age.

**Findings:**

Legal age of consent below 16 years was associated with an 11.0 percentage points higher coverage of HIV testing (95% confidence interval, CI: 7.2 to 14.8), corresponding to a rate ratio of 1.74 (95% CI: 1.35 to 2.13). HIV testing rate had a stronger association with lower age of consent among females than males. The testing rates differences were 14.0 percentage points (95% CI: 8.6 to 19.4) for females and 6.9 percentage points (95% CI: 1.6 to 12.2) for males (*P*-value for homogeneity = 0.07).

**Conclusion:**

This study provides evidence to support the recent World Health Organization’s recommendations that countries should examine current laws and address age-related barriers to uptake of sexual and reproductive health services.

## Résumé

**Objectif:**

Déterminer le lien entre l'âge légal de consentement et la couverture du dépistage du virus de l'immunodéficience humaine (VIH) chez les adolescents dans des pays à forte prévalence d'infection au VIH.

**Méthodes:**

Nous avons analysé les données fournies par des adolescents âgés de 15 à 18 ans dans le cadre de leur participation à une enquête démographique et de santé ou à une enquête sur l'indicateur du sida entre 2011 et 2016 dans 15 pays d'Afrique subsaharienne. Afin d'obtenir une répartition plus équilibrée des caractéristiques mesurées au niveau des individus et des pays, nous avons utilisé un appariement des coefficients de propension entre les adolescents des pays où les lois relatives à l'âge de consentement étaient plus restrictives par opposition aux pays où elles étaient moins restrictives (≤ 15 ans contre ≥ 16 ans). Nous avons estimé le pourcentage des individus qui avaient déclaré avoir bénéficié d'un dépistage du VIH au cours des 12 derniers mois et comparé les écarts entre ces taux de dépistage chez les adolescents exposés à des lois relatives à l'âge de consentement moins restrictives par opposition aux adolescents exposés à des lois relatives à l'âge de consentement plus restrictives. Nous avons également étudié les modifications des effets en fonction du sexe et de l'âge.

**Résultats:**

L'âge légal de consentement inférieur à 16 ans a été associé à une couverture du dépistage du VIH supérieure de 11,0 points de pourcentage (intervalle de confiance, IC, à 95%: 7,2-14,8), soit un rapport de taux de 1,74 (IC à 95%: 1,35-2,13). Le taux de dépistage du VIH a été plus fortement associé à un âge de consentement plus bas chez les femmes que chez les hommes. L'écart des taux de dépistage était de 14,0 points de pourcentage (IC à 95%: 8,6-19,4) chez les femmes et de 6,9 points de pourcentage (IC à 95%: 1,6-12,2) chez les hommes (valeur *P* pour l'homogénéité = 0,07).

**Conclusion:**

Cette étude fournit des données pour soutenir les récentes recommandations de l'Organisation mondiale de la Santé préconisant que les pays examinent leurs lois actuelles et prennent des mesures à l'égard des obstacles liés à l'âge qui freinent le recours à des services de soins de santé sexuelle et procréative.

## Resumen

**Objetivo:**

Estimar la asociación entre la edad legal de consentimiento y la cobertura de las pruebas del virus de la inmunodeficiencia humana (VIH) en los adolescentes de países con una alta carga de VIH.

**Métodos:**

Se analizaron datos de adolescentes de 15 a 18 años que participaron en encuestas demográficas y de salud o encuestas de indicadores de SIDA entre 2011 y 2016, en 15 países de África subsahariana. Para mejorar el equilibrio en la distribución de las características medidas a nivel individual y nacional, se utilizó la comparación de puntuación de propensión entre adolescentes de países con leyes de edad de consentimiento más restrictivas y menos restrictivas (≤15 años frente a ≥16 años). Se calculó el porcentaje de individuos que declararon haberse hecho la prueba del VIH en los últimos 12 meses y se compararon las diferencias en las tasas de pruebas entre los adolescentes expuestos a leyes de edad de consentimiento más bajas o más altas. También se investigaron las modificaciones de los efectos por sexo y edad.

**Resultados:**

La edad legal de consentimiento por debajo de los 16 años se asoció con una cobertura de 11,0 puntos porcentuales más alta de pruebas de VIH (intervalo de confianza del 95 %, IC: 7,21 a 4,8), lo que corresponde a una relación de tasas del 1,74 (IC del 95 %: 1,35 a 2,13). La tasa de pruebas de VIH tenía una asociación más fuerte con la edad de consentimiento más baja entre las mujeres que entre los hombres. Las diferencias en las tasas de pruebas fueron de 14,0 puntos porcentuales (IC del 95 %): 8,6 a 19,4) para las mujeres y de 6,9 puntos porcentuales (IC del 95 %: 1,6 a 12,2) para los hombres (Valor P para la homogeneidad = 0,07).

**Conclusión:**

Este estudio proporciona pruebas que respaldan las recientes recomendaciones de la Organización Mundial de la Salud de que los países deben revisar las leyes vigentes y abordar los obstáculos relacionados con la edad que impiden el acceso a los servicios de salud sexual y reproductiva.

## ملخص

**الغرض:**

تقدير العلاقة بين السن القانونية للرشد وتغطية اختبار فيروس العوز المناعي البشري (HIV) بين المراهقين في البلدان ذات العبء العالي لفيروس العوز المناعي البشري.

**الطريقة:**

قمنا بتحليل البيانات من المراهقين الذين تتراوح أعمارهم بين 15 و18 سنة، والذين شاركوا في الدراسات الاستقصائية السكانية والصحية أو الدراسات الاستقصائية لمؤشرات الإيدز بين عامي 2011 و2016، في 15 بلداً أفريقيا جنوب الصحراء الكبرى. ولتحسين التوازن في توزيع الخصائص المُعايرة على المستويين الفردي والوطني، قمنا باستخدام مقارنة لدرجة النزوع الطبيعي بين المراهقين في البلدان ذات قوانين سن الرشد الأكثر تقييداً مقابل الأقل تقييدًا (أقل من أو يساوي 15 سنة مقابل أكبر من أو يساوي 16سنة). لقد قدرنا النسبة المئوية للأفراد الذين أبلغوا عن أنفسهم بأنهم أجروا اختبارًا لفيروس نقص المناعة البشرية في الأشهر الـ 12 الماضية وقارنوا الاختلافات في معدلات الاختبار هذه بين المراهقين الذين تعرضوا لقوانين سن الرشد الأدنى مقابل الأعلى. نحن أيضا قم بالتحقيق في تعديلات التأثير حسب الجنس والعمر.

**النتائج:**

ارتبط سن الرشد القانوني دون 16 سنة بتغطية أعلى لاختبار فيروس العوز المناعي البشري بنسبة 11.0 نقطة مئوية (فاصل الثقة 95٪، من 7.2 إلى 14.8)، وهو ما يمثل نسبة معدل 1.74 (فاصل الثقة 95%: 1.35 إلى 2.13). كان لمعدل اختبار فيروس نقص المناعة البشرية ارتباط أقوى مع انخفاض سن الرشد بين الإناث مقارنة بالذكور. كانت فروق معدلات الاختبار 14.0 نقطة مئوية (فاصل الثقة 95٪: 8.6 إلى 19.4) بين الإناث و6.9 نقطة مئوية (فاصل الثقة 95%: 1.6 إلى 12.2) للذكور (1.6/12.2) للذكور (القيمة P للتجانس = 0.07).

**الاستنتاج:**

تقدم هذه الدراسة أدلة تدعم توصيات منظمة الصحة العالمية الأخيرة بأن على البلدان دراسة القوانين الحالية ومعالجة العوائق المرتبطة بالعمر في سبيل الحصول على خدمات الصحة الجنسية والإنجابية. ****

## 摘要

**目的:**

旨在探讨艾滋病毒负荷高的国家青少年的法定同意年龄与人类免疫缺陷病毒（即艾滋病毒，HIV）检测覆盖率之间的关系。

**方法:**

我们分析了 2011 年至 2016 年位于 15 个撒哈拉以南非洲国家年龄在 15 至 18 岁的青少年参与人口健康调查或艾滋病指标调查的数据。为了改善衡量个人和国家层面特征的分布平衡，我们采用倾向得分匹配法对最低合法性交年龄限制较多与较少的国家的青少年进行对比研究（≤15 岁组对≥16 岁组）。我们评估了过去 12 个月内个人自行报告已参与艾滋病毒检测的百分比，并对与最低合法性交年龄较高和较低的国家的青少年的检测率差值进行对比。我们还调查了不同性别和年龄带来的影响变化。

**结果:**

最低合法性交年龄（16 岁以下）的青少年与艾滋病毒检测覆盖率相比高出 11.0 个百分点（95% 置信区间 CI：7.2 至 14.8），相当于 1.74 的比率（95% 置信区间 CI：1.35 至 2.13)。与参与检测的最低合法性交年龄较低的男性相比，艾滋病毒检测率与女性的联系更大。女性的检测率差值为 14.0 个百分点（95% 置信区间 CI：8.6 比 19.4），男性为 6.9 个百分点（95% 置信区间 CI：1.6 比 12.2），（同质性的 *P* 值等于 0.07）。

**结论:**

本研究提供的证据支持世界卫生组织最近提出的建议，即各国应审查现行法律，并解决性健康和生殖健康服务在年龄方面的障碍。

## Резюме

**Цель:**

Оценить взаимосвязь между законодательством относительно возраста наступления брачного совершеннолетия и охватом тестирования на вирус иммунодефицита человека (ВИЧ) среди подростков в странах с высоким бременем ВИЧ-инфекции.

**Методы:**

Авторы проанализировали данные, полученные от подростков в возрасте 15–18 лет, которые участвовали в обследованиях демографического положения и состояния здоровья населения или в обследованиях показателей распространенности СПИДа в период с 2011 по 2016 год, в 15 странах Африки к югу от Сахары. Для того чтобы улучшить баланс в распределении полученных характеристик на индивидуальном уровне и в масштабе страны, было использовано сопоставление предрасположенности среди подростков в странах с более строгим и менее строгим законодательством относительно возраста наступления брачного совершеннолетия (возраст ≤ 15 лет по сравнению с возрастом ≥ 16 лет). Авторы оценили процент лиц, самостоятельно сообщивших о прохождении теста на ВИЧ в течение последних 12 месяцев, и сравнили различия в таких показателях тестирования среди подростков из стран с более строгим и менее строгим законодательством относительно возраста наступления брачного совершеннолетия. Также были исследованы изменения показателей в зависимости от пола и возраста.

**Результаты:**

Официальный возраст наступления брачного совершеннолетия ниже 16 лет был связан с увеличением охвата тестированием на ВИЧ на 11,0 процентного пункта (95%-й ДИ: от 7,2 до 14,8), что соответствует отношению рисков 1,74 (95%-й ДИ: от 1,35 до 2,13). Показатель тестирования на ВИЧ был более тесно связан с более ранним возрастом наступления брачного совершеннолетия среди женщин по сравнению с мужчинами. Различия в показателях тестирования составили 14,0 процентного пункта (95%-й ДИ: от 8,6 до 19,4) для женщин и 6,9 процентного пункта (95%-й ДИ: от 1,6 до 12,2) для мужчин (*P*-значение для однородности = 0,07).

## Introduction

In 2017, an estimated 1.8 million adolescents were living with human immunodeficiency virus (HIV)/acquired immunodeficiency syndrome (AIDS) globally.^1^ Of these, 1.5 million (84%) HIV-infected young people live in sub-Saharan Africa, where AIDS is the leading cause of death among people aged 15–19 years.^2^ Most HIV-infected adolescents acquired HIV through mother-to-child transmission and were not diagnosed during infancy. Others were infected through sexual contact; injecting drugs; or through HIV transmission in health-care settings (e.g. blood transfusions).^3^ In 2017, girls aged 15–19 years in sub-Saharan Africa were nearly three times as likely to be newly infected with HIV than adolescent boys.^1^ Adolescents are a group that have been largely left behind in the global AIDS response and AIDS-related deaths declined between 2000 and 2016 for all age groups except adolescents.^1^ However, this neglected group has recently come into focus and the global consensus is that scaling up effective HIV/AIDS prevention, treatment and care for adolescents, especially girls, is urgently needed.^3^^,^^4^

In 2015, the Joint United Nations Programme on HIV and AIDS and United Nations Children’s Fund launched the All In To End the Adolescent AIDS Epidemic (All In) campaign, which focuses on the 25 countries that contribute to 86% of all new HIV infections in adolescents.^3^ By the year 2020, the campaign aims to reduce new HIV infections among adolescents by at least 75%, reduce AIDS-related deaths by at least 65% and end stigma and discrimination for adolescents living with HIV. Strategies to achieve these targets focus on achieving wider access to effective treatment, holistic prevention programmes that combine behavioural, biomedical and structural prevention strategies and increasing HIV testing among adolescents. Specifically, the campaign aims for 90% of adolescents living with HIV to know their status.^3^

The World Health Organization (WHO) recommends HIV testing and counselling, with linkage to prevention, treatment and care, for all adolescents living in generalized epidemic settings, defined as countries where HIV prevalence is consistently over 1% among pregnant women.^5^ However, according to recent data for eastern and southern Africa, only an estimated 23% of girls and 16% of boys aged 15–19 years report being tested for HIV and receiving the result in the past 12 months.^2^ Well-researched barriers to adolescent HIV testing and counselling include difficulties in accessing testing services, fear of discrimination and family reaction and fear of a positive diagnosis and AIDS-related illness or death.^5^ In some countries, adolescents also face legal and policy barriers to HIV testing and counselling, in particular those related to requirements for parent or guardian consent to access HIV testing and counselling services.^6^ Restrictive age-of-consent legislation is acknowledged as a potential barrier to HIV testing among adolescents and there is an increasing interest to review and reform age-of-consent laws.^3^^,^^5^

According to a review of age-of-consent laws in African countries, 19 of the 33 countries had clear national laws supporting independent HIV testing and counselling for people younger than 18 years.^7^ Most African countries set the age of consent at 16 or 18 years; however, some countries like South Africa and Uganda permit independent access to HIV testing and counselling services as early as 12 years of age.^5^ The intent of policies on age of consent is to protect youth minors; yet, requiring parental consent to access HIV testing and counselling may be a barrier to such services.^8^^–^^10^ In particular, parental consent requirements may deter young women from accessing important sexual and reproductive health services, including HIV testing and counselling, due to fear of disclosure or violence.^9^^,^^11^^,^^12^ Healthy parental involvement and communication have been shown to have positive effects on youth decision-making around sexual and reproductive health issues,^8^ however, there is little evidence to suggest that parental consent fosters positive parental involvement.^7^

The review on age-of-consent laws recommended that countries consider setting the minimum legal age of consent for HIV testing and counselling to 12 or 14 years of age, but the authors acknowledge there are little data to support this recommendation.^7^ Although several countries in sub-Saharan Africa have lowered the legal age of consent in the past decade, little is known about the impact of these changes. Several stakeholders have called for more research to understand the effects of lowering the legal age to consent to HIV testing and counselling.^7^^,^^13^ The objective of this study was to estimate the association between legal age of consent and the rates of HIV testing among adolescents, using a quasi-experimental approach and data from 15 sub-Saharan countries.

## Methods

### Study design and measures

This is a cross-national study to estimate the association between legal age of consent and coverage of HIV testing and counselling among adolescents aged 15–18 years. We used a cut-off of 18 years because individuals older than this age were eligible to consent to HIV testing and counselling across all countries. We used a quasi-experimental propensity score matching approach to achieve balance in the distribution of measured covariates between countries with more and less restrictive consent legislation. We employed this approach expecting that factors such as national HIV prevalence would confound the relationship between age-of-consent policies and uptake of HIV testing among adolescents, as countries with higher HIV prevalence tend to have higher coverage of HIV testing and counselling. These countries may have also lowered the legal age of consent in response to the country’s HIV epidemic.^6^ By matching the groups being compared for variables that might predict the likelihood of exposure to less restrictive age-of-consent laws, the propensity score approach can improve control of confounding factors and reduce bias.^14^

We used nationally representative household survey data from Demographic and Health Surveys (DHS) and Aids Indicator Surveys.^15^ We included Sub-Saharan African countries targeted in the All In campaign^3^ with at least one nationally-representative survey, conducted between 2011 and 2016, that asked participants whether they had received an HIV test within the past 12 months. Adolescents aged 15–18 years and who had not previously given birth and were not currently pregnant, were eligible for the study. We set these inclusion criteria as HIV testing is frequently linked to antenatal care services regardless of consent laws.^16^

We linked individual-level data on self-reported HIV testing and other covariates to information on national age-of-consent laws for independent HIV testing and counselling. We obtained information about such laws from a review conducted in 2011 and 2012.^7^ We also reviewed national policy documents to ensure each survey data set were matching the correct legislation in place at the time of the survey.

### Statistical analysis

**Fig. 1:**
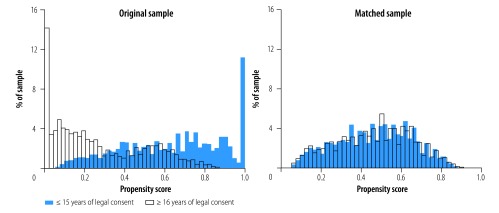
The estimated propensity scores by legal age of consent to independent human immunodeficiency virus testing and counselling for the original and matched samples, 15 sub-Saharan countries, 2011–2016

Notes: We used a caliper of 0.01 for the matched samples. Countries included in the study are listed in Table 1.

We used 1–1 nearest neighbour propensity score matching without replacement and tested different caliper sizes to ensure that potential confounders were balanced between adolescents in the two groups, that is, age-of-consent policy ≤ 15 years versus ≥ 16 years. We identified potential country- and individual level confounders a priori. The four country-level variables were: (i) HIV prevalence: (ii) adolescent fertility rate; (iii) health expenditures per capita; and (iv) comprehensive HIV knowledge among youth, and the seven individual-level variables were: (i) age; (ii) sex; (iii) marital status; (iv) education; (v) rural residence; (vi) previous sexual activity; and (vii) assets-based household wealth. We selected a caliper size of 0.01 after comparing changes in mean standardized bias and sample size (comparison available from the corresponding author). Histograms showing the estimated propensity scores by legal age of consent for HIV testing and counselling for the original and matched samples are presented in Fig. 1.

Using the propensity-score-matched sample, we assessed the association between age-of-consent polices and adolescent HIV testing with logistic regression models. To facilitate interpretation and reporting of associations on the absolute probability scale, we calculated rate ratios (RR) for the proportion of people tested for HIV in the past 12 months and rate differences from average marginal probabilities estimated from the regression coefficients.^17^ In this primary analysis we included all adolescents, since more adolescents in sub-Saharan Africa have been infected through mother-to-child transmission than through sexual contact. Furthermore, the WHO’s recommendations state that all adolescents should receive HIV testing and counselling in generalized epidemic settings.^5^ We conducted a second analysis restricted only to those who reported ever having had sex.

We also investigated whether the association between age-of-consent policies and HIV testing rates differed by sex or age (15–16 years versus17–18 years of age), by including interaction terms between these variables and the age-of-consent policy. A *χ^2^* test for formal statistical comparison of homogeneity of the estimated RRs and rate differences was used to assess whether the associations differed by sex or age.^18^ Given the low statistical power of homogeneity tests, we considered more liberal *P*-value cut-offs of 0.10 to be suggestive of potential effect modification.^19^ We accounted all analyses for clustering at the country level and conducted the analyses using Stata version 14.1 (StataCorp LCC, College Station, United States of America).

## Results

The full sample included 62 628 adolescents, of which 39 339 were females and 23 289 were males. The legal age of consent for independent HIV testing and counselling was ≤ 15 years in six of the countries and ≥ 16 years in the remaining nine countries (Table 1). Table 1 also presents estimated coverage of HIV testing in the past 12 months for female and male adolescents. Table 2 presents characteristics of the sample stratified by age of consent. The HIV prevalence did not differ between countries with more or less restrictive age-of-consent policies (7.1% in countries with ≥ 16 years as age consent and 6.9% in countries with ≤ 15 years). The two groups were similar on other characteristics, except for level of education and rural residence. In countries with ≥ 16 years as the age of consent, 59% of the adolescents had some secondary schooling (95% CI: 48 to 69%) versus 36% (95% CI: 28 to 44%) in countries with < 16 years as the age of consent.

**Table 1:** Characteristics of the countries and surveys used in the study on national age-of-consent laws and adolescent human immunodeficiency virus testing, 15 sub-Saharan countries, 2011–2016

Country	Survey year(s) and data source	Sample size (adolescents aged 15–18 years)	Legal age of consent for HTC, years^a^	Estimated HIV prevalence for survey year, %^b^	% of adolescents tested for HIV in the past 12 months^c^	
					Female	Male
Cameroon	2011 DHS	3820	18	4.5	4.8	5.6
Côte d’Ivoire	2011 DHS	4184	18	3.6	8.3	5.2
Democratic Republic of the Congo	2013 DHS	2075	18	0.9	3.6	1.3
Ethiopia	2011 DHS	4346	15	1.3	22.9	5.6
Kenya	2014 DHS	6863	15	5.7	31.1	27.3
Lesotho	2014 DHS	1699	12	24.7	32.8	30.4
Malawi	2015–2016 DHS	5179	13	9.5	23.7	24.3
Mozambique	2011 DHS	2730	16	13.8	9.5	6.6
Namibia	2013 DHS	2025	16	14.1	22.7	15.0
Nigeria	2013 DHS	9114	18	3.1	3.5	2.3
Rwanda	2014–2015 DHS	3298	15	3.2	30.0	26.0
Uganda	2011 DHS, 2011 AIS	5394	12	7.3	26.4	16.6
United Republic of Tanzania	2011AIS	3496	18	5.4	16.8	13.2
Zambia	2013 DHS	5154	16	12.7	25.0	22.1
Zimbabwe	2015 DHS	3251	16	13.9	24.8	21.1

AIS: Aids Indicator Survey; DHS: Demographic and Health Survey; HIV: human immunodeficiency virus; HTC: human immunodeficiency virus testing and counselling.

^a^ We obtained the data from the World Health Organization.^5^

^b^ HIV prevalence among adults age 15–49 years.^20^

^c^ For adolescents aged 15–18 years. Estimated from current DHS study sample, proportions are weighted by probability of selection weights.

**Table 2:** Sample characteristics by legal age of consent to independent human immunodeficiency virus testing and counselling, 15 sub-Saharan countries, 2011–2016

Characteristic	Legal age of consent, mean value (95% CI)	
	≤ 15 years *n* = 26 779 adolescents	≥ 16 years *n* = 35 849 adolescents
**Individual-level variable**		
Age, years	16.39 (16.35 to 16.43)	16.41 (16.37 to 16.44)
% of females	67 (63 to 71)	60 (54 to 67)
% of never married adolescents	94 (92 to 97)	92 (88 to 95)
% of adolescents who attended secondary school	36 (28 to 44)	59 (48 to 69)
% of adolescents residing in rural areas	76 (69 to 82)	57 (50 to 63)
Household wealth quintile, %		
Poorest	16 (14 to 18)	15 (14 to 16)
Poorer	19 (16 to 21)	17 (16 to 19)
Middle	20 (18 to 22)	21 (20 to 22)
Richer	22 (21 to 24)	22 (21 to 23)
Richest	23 (19 to 27)	25 (22 to 28)
% of adolescents who ever had sexual interaction	28 (21 to 35)	33 (26 to 40)
**Country-level variable**		
HIV prevalence, %^a^	6.9 (3.2 to 10.5)	7.1 (3.1 to 11.0)
Adolescent fertility rate, births per 1000 women^b^	96 (65 to 128)	117 (106 to 128)
Health expenditure per capita, PPP in Int$^c^	141 (98 to 184)	176 (79 to 273)
Comprehensive HIV knowledge among youth, %^d^	44 (33 to 55)	32 (21 to 42)
No. of countries	6	9

CI: confidence interval; HIV: human immunodeficiency virus; Int$: international dollars.

^a^ HIV prevalence among adults age 15–49 years.^20^

^b^ Women aged 10–19 years.^21^

^c^ Purchasing power parity in constant 2011 Int$.^21^

^d^ Percentage women and men aged 15–24 years, who correctly identify the two major ways of preventing the sexual transmission of HIV (using condoms and limiting sex to one faithful, uninfected partner), who reject the two most common local misconceptions about HIV transmission, and who know that a healthy-looking person can have HIV.^22^

Notes: The individual-level variables are weighted values. We obtained survey data for 15 countries, presented in Table 1, from Demographic and Health Surveys and AIDS Indicator Surveys.

In the propensity score-matched sample, lower age of consent (≤ 15 years versus ≥ 16 years) was associated with a 11.0-percentage point higher coverage of HIV testing in the past 12 months (95% CI: 7.2 to 14.8; Table 3). This difference corresponds to a RR of 1.74 (95% CI: 1.35 to 2.13). There were also differences in the coverage of HIV testing by sex and age. Females were more likely to have been tested for HIV than males (rate difference: 8.6 percentage points; 95% CI: 4.2 to 13.1). As expected, HIV testing increased with age, coverage was 9.6 percentage points higher (95% CI: 7.7 to 11.6) among adolescents aged 18 than 15 years. In the analysis restricted to sexually active adolescents, the rate difference between lower age of consent versus higher was similar to the full sample (11.8 percentage points; 95% CI: 7.0 to 16.6), while the RR was slightly smaller (1.52; 95% CI: 1.21 to 1.83).

**Table 3:** Association between national age of consent and human immunodeficiency virus testing and counselling, 15 sub-Saharan countries, 2011–2016

Characteristic	HIV testing in the past 12 months			
	All adolescents (*n* = 30 652)^a^		Sexually active adolescents (*n* = 10 561)^a^	
	Rate difference, percentage points (95% CI)	RR (95% CI)	Rate difference, percentage points (95% CI)	RR(95% CI)
Legal age of consent				
≥ 16 years	Ref.	Ref.	Ref.	Ref.
≤ 15 years	11.0 (7.2 to 14.8)	1.74 (1.35 to 2.13)	11.8 (7.0 to 16.6)	1.52 (1.21 to 1.83)
**Sex**				
Male	Ref.	Ref.	Ref.	Ref.
Female^b^	8.6 (4.2 to 13.1)	1.55 (1.19 to 1.91)	12.1 (8.0 to 16.2)	1.54 (1.30 to 1.78)
**Age of respondent**				
15 years	Ref.	Ref.	Ref.	Ref.
16 years	3.6 (2.2 to 5.0)	1.24 (1.14 to 1.34)	2.3 (−1.4 to 6.0)	1.10 (0.93 to 1.26)
17 years	6.3 (4.7 to 7.9)	1.42 (1.30 to 1.53)	5.7 (0.6 to 10.8)	1.24 (1.00 to 1.48)
18 years	9.6 (7.7 to 11.6)	1.64 (1.45 to 1.82)	8.7 (5.2 to 12.2)	1.37 (1.19 to 1.56)

CI: confidence interval; HIV: human immunodeficiency virus; Ref.: reference group; RR: rate ratio.

^a^ Adolescents aged 15–18 years.

^b^ Never pregnant females aged 15–18 years.

Notes: We obtained survey data from Demographic and Health Surveys and AIDS Indicator Surveys. We formed propensity score matched sample by matching adolescents exposed to either ≤ 15 or ≥ 16 years of age-of-consent law, on individual, household and national-level characteristics (Table 1), using 1:1 nearest neighbour matching.

Examining variables that could potential modify the association between legal age of consent and HIV testing rate showed that the increased coverage seen in lower legal age-of-consent group is greater among females than males (rate difference:14.0 for females and 6.9 for males; *P*-value for homogeneity: 0.07). Among adolescents who reported being sexually active, the association between age of consent and testing rate by sex was slightly attenuated (*P*-value for homogeneity: 0.24). There was no evidence that associations between age of consent and HIV testing varied by age, either in the full or sexually active sample (*P*-value for homogeneity: 0.83 and  0.66, respectively; Table 4).

**Table 4:** Variables modifying the association between legal age of consent and adolescent human immunodeficiency virus testing coverage, 15 sub-Saharan countries, 2011–2016

Group	HIV testing rate in the past 12 months, weighted % (95% CI)^a^		Difference in testing rate (95% CI)	*P*^b^
	Age of consent ≤ 15 years	Age of consent ≥ 16 years		
**Adolescents (*n* = 30 652)**				
Sex				
Female	31.3 (24.9 to 37.7)	17.3 (11.6 to 23.0)	14.0 (8.6 to 19.4)	0.07
Male	18.8 (12.3 to 25.3)	11.9 (7.6 to 16.2)	6.9 (1.6 to 12.2)	
Age group				
15–16 years	22.8 (17.9 to 27.6)	12.0 (7.6 to 16.4)	10.7 (7.7 to 13.8)	0.83
17–18 years	28.9 (12.2 to 22.9)	17.6 (12.2 to 22.9)	11.3 (7.0 to 15.7)	
**Sexually active adolescents (*n* = 10 561)**				
Sex				
Female	40.9 (31.6 to 50.1)	27.0 (18.2 to 35.9)	13.9 (7.8 to 19.9)	0.24
Male	26.6 (19.4 to 33.9)	17.5 (11.3 to 23.8)	9.1 (3.8 to 14.4)	
Age group				
15–16 years	30.2 (22.8 to 37.5)	19.4 (12.9 to 26.0)	10.7 (6.4 to 15.0)	0.66
17–18 years	36.8 (28.5 to 45.1)	24.5 (16.3 to 32.6)	12.4 (6.1 to 18.7)	

^a^ Adolescents aged 15–18 years.

^b^ *P*-value for homogeneity of the differences in coverage. We used χ^2^ test.

Notes: We obtained survey data for 15 countries, presented in Table 1, from Demographic and Health Surveys and AIDS Indicator Surveys.

## Discussion

This study shows that a lower legal age of consent to independent HIV testing and counselling is associated with an increase in HIV testing rate among adolescents in high-HIV burden countries. To date, recommendations to legally lower the age of consent for HIV/AIDS services, such as those in the WHO guidance document^5^ have been based on an awareness of the ethical importance of supporting the notion of adolescents’ evolving capacities.^23^ Such a notion has been delineated in the International Convention on the Rights of the Child.^24^ The recommendations were also based on the recognition of the pragmatic and psychosocial barriers to service uptake that age-of-consent laws may introduce. The results of this study provide evidence to support these recommendations.

Age-of-consent laws can be a barrier to adolescents accessing relevant HIV/AIDS services.^10^ This study provides new evidence that a lower legal age of consent could address this barrier, especially in countries with more restrictive legislation, and increase HIV testing and counselling uptake. HIV testing and counselling uptake among adolescents, in turn, has been associated with lower incidence of HIV infection over time.^25^ If, as the findings of this study suggest, lower age-of-consent laws can be an upstream facilitator for improving access to HIV testing and counselling, such laws may also potentially indirectly act to reduce HIV incidence rates.

The results presented here indicate a stronger association between lower age-of-consent laws and HIV testing rate among females than males. Such a finding suggests that lowering the legal age of consent could have a greater effect on adolescent girls, who are more affected by the HIV epidemic. Adolescent girls are at greater risk, both since they have a higher biological susceptibility to HIV infection and because of sociodemographic characteristics and sociocultural beliefs and practices that deter them from accessing sexual and reproductive health services.^26^ With little evidence on effective HIV prevention interventions to protect young women,^27^ this study provides a potentially important finding that is well aligned with current efforts to promote gender equality, as articulated by sustainable development goal 5.^28^

This study has several strengths. We used comparable high-quality data from the well-established DHS program for 15 of the18 high-HIV burden countries in sub-Saharan Africa. We relied on a comprehensive review of age-of-consent laws conducted by WHO. We employed propensity score matching to minimize potential confounding by individual- and country-level factors, including national HIV prevalence and knowledge of HIV. Changes in HIV testing uptake in young people have been attributed to several factors, including access to testing facilities, perceived attitudes of staff towards young people and perceptions around confidentiality.^29^ We therefore matched factors likely to be influenced by other policy and programme efforts that affect access and barriers to HIV testing and counselling among adolescents, and that may coincide with age-of-consent laws.

The study also has limitations. The propensity score analysis does not account for potential unmeasured confounding factors. Therefore, there may be factors related to a country’s sociopolitical acceptance of adolescent sexuality and autonomy, which could influence both the adoption of lower age-of-consent laws and uptake of HIV testing and counselling for reasons other than age-related legal barriers to care. For example, in 2014, the East and Southern Africa Regional Office of the United Nations Population Fund, in collaboration with the Africa Regional Office of the International Planned Parenthood Federation, launched a multi-year initiative to scale up adolescent and youth-friendly health services in the region.^30^ Recent assessment of the implementation of such services revealesd significant variation across and within countries, however data on the specific implementation successes or gaps by country are not yet available. However, youth-friendly health services could have so far an undetermined relationship with age-of-consent laws and adolescent HIV testing and counselling uptake. Potential mechanisms for such a relationship could be that these services enhanced quality of care, improved provider knowledge of the legal rights of adolescents, or increased demand for services.

Another limitation is the reliance on self-reports of HIV testing, which may be affected by recall and/or reporting bias.^31^ HIV testing may be either over-reported, if being tested is regarded as the responsible thing to do, or underreported, if getting tested for HIV is perceived as admitting to a socially undesirable behaviour (e.g. premarital sex).^29^ Evidence on the likelihood and possible magnitude of these two potential reporting bias scenarios is limited. Although plausible, we have no reason to believe the tendency to under- or over-report HIV testing in a confidential survey would differ systematically according to age-of-consent laws. Furthermore, we note that the 15 countries included in the analysis had five different legal ages of consent, varying between 12 and 18 years. From an analytical standpoint, we were not able to examine differences among these different legal ages and elected to compare laws stipulating ≤ 15 years versus ≥ 16 years. We based this choice on WHO recommendations to consider setting the minimum legal age-of-consent for HIV testing and counselling to 12 or 14 years, as opposed to most countries that set a minimum legal age at 16 or 18.^7^ The results in this study should thus be interpreted as a comparison between countries with lower versus higher legal ages of consent, and not as a comparison between specific legal ages.

This study provides evidence that lowering the legal age of consent to HIV testing and counselling may be a potential mechanism for increasing coverages of HIV testing among adolescents in high-HIV burden countries. The findings support WHO’s recommendations that countries should examine current laws and address age-related barriers to uptake of sexual and reproductive health services. Additional research would help elucidate any negative or unintended consequences of lowering the legal age of consent for independent HIV testing and counselling.

Removing legal barriers represents one component of a comprehensive strategy of the All In campaign to tackle the underlying causes of risk and vulnerability among adolescents. This strategy will help to achieve the campaign’s goals of significantly reducing new HIV infections and AIDS-related deaths among adolescents and ensuring that 90% of adolescents living with HIV know their status.

## References

[1] Children and AIDS: statistical update. New York: United Nations Children’s Fund; 2017. Available from: https://data.unicef.org/topic/hivaids/global-regional-trends [cited 2018 Nov 8].

[2] For every child, end AIDS: seventh stocktaking report, 2016. New York: United Nations Children’s Fund; 2016. Available from: https://data.unicef.org/wp-content/uploads/2016/12/HIV-and-AIDS-2016-Seventh-Stocktaking-Report.pdf [cited 2018 Feb 1].

[3] All in to end the adolescent AIDS epidemic. . New York: United Nations Children’s Fund; 2015. Available from: http://www.unaids.org/sites/default/files/media_asset/ALLIN2016ProgressReport_en.pdf[cited 2018 Feb 1].

[4] The Gap report. Geneva: UN Joint Programme on HIV/AIDS; 2014. Available from: http://www.unaids.org/en/resources/campaigns/2014/2014gapreport/gapreport[cited 2018 Jan DAY].

[5] HIV and adolescents: guidance for HIV testing and counselling and care for adolescents living with HIV: recommendations for a public health approach and considerations for policy-makers and managers. Geneva: World Health Organization; 2013. Available from: http://www.who.int/hiv/pub/guidelines/adolescents/en/[cited 2018 Feb 1]. 25032477

[6] Sam-Agudu NA, Folayan MO, Ezeanolue EE. Seeking wider access to HIV testing for adolescents in sub-Saharan Africa. Pediatr Res. 2016 6;79(6):838–45. 10.1038/pr.2016.28 26882367

[7] Fox K, Ferguson J, Ajose W, Singh J, Marum E, Baggaley R. HIV and adolescents: guidance for HIV testing and counselling and care for adolescents living with HIV. Annex 15: Adolescent consent to testing: a review of current policies and issues in sub-Saharan Africa. Geneva: World Health Organization; 2013. Available from: http://apps.who.int/iris/bitstream/10665/95147/1/WHO_HIV_2013.141_eng.pdf[cited 2018 Feb 1].

[8] Denison J, Lungu N, Dunnett-Dagg WA, McCauley A, Sweat MD. Social relationships and adolescents’ HIV counseling and testing decisions in Zambia. Washington, DC: Horizons; 2006. Available from: https://pdfs.semanticscholar.org/e0cf/bc51b908ab0b0ff55b19c1d68a33ac0f800a.pdf[cited 2018 Jan 1].

[9] Jackson S, Hafemeister TL. Impact of parental consent and notification policies on the decisions of adolescents to be tested for HIV. J Adolesc Health. 2001 8;29(2):81–93. 10.1016/S1054-139X(00)00178-6 11472866

[10] The voices, values and preference of adolescents on HIV testing and counselling: consultation for the development of the World Health Organization HIV testing and counselling guidelines for adolescents. Geneva: World Health Organization; 2013. Available from: http://www.who.int/iris/handle/10665/95143[cited 2018 Mar 1].

[11] Meehan TM, Hansen H, Klein WC. The impact of parental consent on the HIV testing of minors. Am J Public Health. 1997 8;87(8):1338–41. 10.2105/AJPH.87.8.1338 9279271 PMC1381096

[12] Reddy DM, Fleming R, Swain C. Effect of mandatory parental notification on adolescent girls’ use of sexual health care services. JAMA. 2002 8 14;288(6):710–4. 10.1001/jama.288.6.710 12169074

[13] Asaolu IO, Gunn JK, Center KE, Koss MP, Iwelunmor JI, Ehiri JE. Predictors of HIV testing among youth in sub-Saharan Africa: a cross-sectional study. PLoS One. 2016 10 5;11(10):e0164052. 10.1371/journal.pone.0164052 27706252 PMC5051677

[14] Oakes JM, Johnson PJ. Propensity score matching for social Epidemiology. In: Oakes JM, Kaufman JS, editors. Methods in social epidemiology. San Francisco: John Wiley & Sons; 2016.

[15] Survey types [internet]. Rockville: ICF; 2018. Available from: https://dhsprogram.com/What-We-Do/Survey-Types/index.cfm [cited 2018 Feb 1].

[16] Idele P, Gillespie A, Porth T, Suzuki C, Mahy M, Kasedde S, et al. Epidemiology of HIV and AIDS among adolescents: current status, inequities, and data gaps. J Acquir Immune Defic Syndr. 2014 7 1;66 Suppl 2:S144–53. 10.1097/QAI.0000000000000176 24918590

[17] Muller CJ, MacLehose RF. Estimating predicted probabilities from logistic regression: different methods correspond to different target populations. Int J Epidemiol. 2014 6;43(3):962–70. 10.1093/ije/dyu029 24603316 PMC4052139

[18] Kaufman JS, MacLehose RF. Which of these things is not like the others? Cancer. 2013 12 15;119(24):4216–22. 10.1002/cncr.28359 24022386 PMC4026206

[19] Fletcher J. What is heterogeneity and is it important? BMJ. 2007 1 13;334(7584):94–6. 10.1136/bmj.39057.406644.68 17218716 PMC1767262

[20] AIDSinfo [internet]. Geneva: UN Joint Programme on HIV/AIDS; 2018. Available from: http://aidsinfo.unaids.org/ [cited 2018 Nov 9].

[21] World Bank Open Data [internet]. Washington, DC: World Bank; 2018. Available from: https://data.worldbank.org [cited 2018 Feb 1].

[22] STATCompiler. The DHS Program. Rockville: ICF; 2018. Available from: https://www.statcompiler.com/en/ [cited 2018 Mar 1].

[23] Cook RJ, Erdman JN, Dickens BM. Respecting adolescents’ confidentiality and reproductive and sexual choices. Int J Gynaecol Obstet. 2007 8;98(2):182–7. 10.1016/j.ijgo.2007.04.018 17582416

[24] Convention on the Rights of the Child. Geneva: Office of the United Nations High Commissioner; 1996–2018. Available from: https://www.ohchr.org/Documents/ProfessionalInterest/crc.pdf [cited 2018 Jan 1].

[25] Rosenberg NE, Westreich D, Bärnighausen T, Miller WC, Behets F, Maman S, et al. Assessing the effect of HIV counselling and testing on HIV acquisition among South African youth. AIDS. 2013 11 13;27(17):2765–73. 10.1097/01.aids.0000432454.68357.6a 23887069 PMC4028633

[26] Dellar RC, Dlamini S, Karim QA. Adolescent girls and young women: key populations for HIV epidemic control. J Int AIDS Soc. 2015 2 26;18(2) Suppl 1:19408. 25724504 10.7448/IAS.18.2.19408 PMC4344544

[27] Abdool Karim Q, Dellar R. Inclusion of adolescent girls in HIV prevention research - an imperative for an AIDS-free generation. J Int AIDS Soc. 2014 3 8;17(1):19075. 10.7448/IAS.17.1.19075 24629847 PMC3955761

[28] The sustainable development goals report 2018. New York: United Nations; 2015. Available from: https://unstats.un.org/sdgs/files/report/2018/TheSustainableDevelopmentGoalsReport2018-EN.pdf [cited 2018 Feb 1].

[29] Global AIDS monitoring 2017. Geneva: UN Joint Programme on HIV/AIDS; 2016. Available from: http://www.aidsdatahub.org/sites/default/files/highlight-reference/document/UNAIDS_2017_Global_AIDS_Monitoring_2016.pdf [cited 2018 Mar DAY].

[30] Assessment of adolescent and youth-friendly health service delivery in the east and southern Africa Region. Sunninghill: United Nations Population Fund, East and Southern Africa Regional Office; 2017. Available from: https://esaro.unfpa.org/sites/default/files/pub-pdf/Research%20Summary%20-%20Assessment%20of%20Adolescent%20and%20Youth-Friendly%20Health_0.pdf [cited 2018 Mar 1].

[31] Brown JL, DiClemente RJ. The need for biological outcomes to complement self-report in adolescent research. Pediatrics. 2015 9;136(3):e551–3. 10.1542/peds.2015-0239 26283780 PMC4552089

